# Gender differences in cognitive Theory of Mind revealed by transcranial direct current stimulation on medial prefrontal cortex

**DOI:** 10.1038/srep41219

**Published:** 2017-01-24

**Authors:** Mauro Adenzato, Michela Brambilla, Rosa Manenti, Lucia De Lucia, Luigi Trojano, Sara Garofalo, Ivan Enrici, Maria Cotelli

**Affiliations:** 1 Department of Psychology, University of Turin, Turin, Italy; 2 Center for Cognitive Science, University of Turin, Turin, Italy; 3 Neuroscience Institute of Turin, Turin, Italy; 4 Neuropsychology Unit, IRCCS Centro San Giovanni di Dio Fatebenefratelli, Brescia, Italy; 5 Department of Psychology, Second University of Naples, Caserta, Italy; 6 Department of Psychiatry, University of Cambridge, Cambridge, UK; 7 Department of Philosophy and Educational Sciences, University of Turin, Turin, Italy

## Abstract

Gender differences in social cognition are a long discussed issue, in particular those concerning Theory of Mind (ToM), i.e., the ability to explain and predict other people’s mental states. The aim of this randomized, double-blind, placebo-controlled study was to test the hypothesis that anodal tDCS over the medial prefrontal cortex (mPFC) selectively enhances cognitive ToM performance in females. In the first experiment we administered to sixteen females and sixteen males a cognitive ToM task during anodal or placebo tDCS over the mPFC. In the second experiment further sixteen females completed the task receiving anodal or placebo tDCS over the vertex. The results showed that anodal tDCS over the mPFC enhances ToM in females but not in males, an effect indicated by enhanced ToM in females that received anodal tDCS over the mPFC compared with females that received tDCS over the vertex. These findings are relevant for three reasons. First, we found evidence of gender-related differences in cognitive ToM, extending previous findings concerning affective ToM. Second, these differences emerge with anodal stimulation of the mPFC, confirming the crucial role of this area in cognitive ToM. Third, we show that taking into account gender-related differences is mandatory for the investigation of ToM.

Theory of Mind (ToM) is the social cognitive ability to explain and predict other people’s actions in terms of the underlying mental states, such as beliefs, intentions, or feelings^1^. For example, by means of ToM, we interpret another person reaching towards a glass of water in terms of an intention to drink, rather than in terms of the mechanical forces used in such an action, or we recognize the intended meaning underlying an ironic remark^2^. ToM is thought to be at the core of any successful social interactions, and its impairment has been implicated in various neuropsychiatric disorders involving altered social understanding^3^,^4^,^5^.

Gender differences in social cognitive processes are a long discussed issue, in particular those concerning ToM. The main theoretical framework in this respect is the empathizing/systematizing theory of psychological sex differences proposed by Baron-Cohen^6^, according to which females are, on average, more disposed to an empathizing style— i.e., the drive to identify others’ mental states in order to predict their behavior and respond with an appropriate emotion. On the other hand, males are, on average, more disposed to a systematizing style, i.e., the drive to predict and to respond to the behavior of non-agentive deterministic systems by inferring the rules that govern such systems. This theory has been supported by behavioral studies showing that female subjects, compared to their male counterparts, score higher on tests related to the affective dimension of social cognition, such as emotion recognition^7^, social sensitivity^8^, empathy^9^, and emotional intelligence^10^. It has been hypothesized that the greater sensitivity of females for affective social stimuli is an ancient biological phenomenon, mainly due to a series of evolutionary changes in females’ capacity to detect and respond adaptively to newborns’ signals and need for a prolonged postnatal period^11^. Accordingly, neuroimaging studies found differences between females and males in tasks involving the evaluation of affective scenes^12^, empathic face-to-face interactions^13^, humor appreciation^14^, social reputation in pain perception^15^, and social appraisals^16^.

The ability to attribute mental states to ourselves and others has been proposed to be based on a distributed neural network, including the complex formed by the right and left temporo-parietal junctions (TPJs), the precuneus, and the medial prefrontal cortex (mPFC)^17^. Several studies have suggested the pivotal role of the mPFC in ToM abilities (see ref. 18 for a review), and findings suggest that while the dorsolateral and the ventromedial PFCs exhibit preferences for the processing of cognitive (e.g., intentions, beliefs about beliefs) and affective (e.g., emotions, beliefs about feelings) mental states, respectively, the posterior regions of the ToM neural network (i.e., the precuneus and TPJs) do not exhibit this marked preference but play a major role in assigning agency to these mental states^19^,^20^.

Brain stimulation techniques have confirmed that ToM could be considered a multidimensional construct, and that different brain regions are differentially recruited during cognitive and affective ToM tasks^21^,^22^,^23^,^24^. To the best of our knowledge, to date, only two studies applied brain stimulation to investigate gender differences in the social cognitive domain^25^,^26^. In particular, both studies used transcranial direct-current stimulation (tDCS)—a safe, noninvasive brain stimulation technique in which electrical current is directly applied to the head to generate an electrical field that modulates neuronal activity^27^,^28^. Anodal tDCS has a general facilitation effect, whereas cathodal tDCS has a general inhibitory effect. Using tDCS, Conson and colleagues^25^ compared the effects of stimulation of the dorsolateral PFC between males and females in terms of both visual perspective taking and recognition of emotional facial expressions. Their findings showed that after anodal right/cathodal left stimulation, both males and females have a negative effect on the tendency to adopt another person’s visual perspective, but males are significantly faster in the explicit recognition of fearful facial expressions. Fumagalli and colleagues^26^ investigated utilitarian behavior by means of a moral judgment task and found that tDCS of the ventral PFC influences the evaluation of the advantages and disadvantages of utilitarian decisions in both males and females, albeit to a greater degree in females.

Interestingly, to date, no studies have used tDCS to specifically investigate gender-related differences in ToM. The main aim of the present study was to shed light on this topic. To this end, we used tDCS for the first time in the investigation of gender-related differences in cognitive ToM, extending previous behavioral, neuroimaging, and brain stimulation studies investigating almost exclusively the affective domain. We applied anodal tDCS on the mPFC to modulate healthy participants’ performance on an adapted version of a cognitive ToM task, tapping the ability to represent other people’s intentions from the observation of their daily actions. Considering both the greater sensitivity of females to social stimuli and the role played by the mPFC in ToM, we expected to find gender-related differences in the change induced by tDCS in the performance of cognitive ToM tasks.

## Results

### Experiment 1

#### Demographic and RME data analysis

No significant differences were found between males and females in terms of age (24.2 ± 3.7 and 23.0 ± 3.2 years, respectively; p = 0.45), education (14.8 ± 2.9 and 14.7 ± 2.3, respectively; p = 0.90), Edinburgh Handedness Index (78.2 ± 20.2 and 80.2 ± 21.9, respectively; p = 0.68) and RME test scores (23.6 ± 3.1 and 25.1 ± 3.3, respectively; p = 0.28). Regarding the RME test, the overall group reached a mean of 24.3 ± 3.3 points (range, 20–30) indicating age- and gender-adequate ToM abilities according to the Italian normative data provided by Vellante *et al*.^29^.

#### Attribution of Intentions task

Accuracy analysis. No significant effect for “gender” (F_(1,30)_ = 0.39, p = 0.54, η^2^ = 0.001), type of “stimuli” (F_(1,30)_ = 0.59, p = 0.45, η^2^ = 0.02), type of “stimulation” (F_(1,30)_ = 0.79, p = 0.38, η^2^ = 0.03), and the interactions between factors were found. A ceiling effect was observed in accuracy for both stimulation conditions (anodal mPFC stimulation: males = 96.5 ± 4.2%, females = 97.2 ± 2.0%, range = 88–100%; placebo stimulation: males = 95.5 ± 3.0%, females = 96.5 ± 2.8%, range = 88–100%).

##### Reaction time analysis

**Figure 1:**
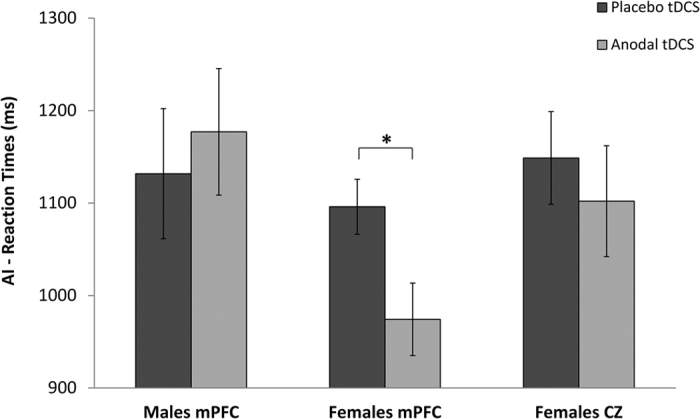
Changes in RTs in the AI task for anodal tDCS and placebo tDCS. Only in the female group that received tDCS over mPFC the RTs were improved after anodal tDCS compared to placebo stimulation. No tDCS effects were shown for the female group that received tDCS over Cz and for the male group that received tDCS over mPFC. No significant differences between the groups were observed in the placebo condition. Asterisk indicates a significant effect (p < 0.05).

RT analysis indicated a significant interaction between “gender” and type of “stimulation” (F_(1,30)_ = 9.31, p = 0.005, η^2^ = 0.24; Fig. 1). The type of “stimuli” (F_(1,30)_ = 0.28, p = 0.60, η^2^ = 0.009), and the interaction between “gender” and type of “stimuli” (F_(2,30)_ = 3.70, p = 0.064, η^2^ = 0.11) and between type of “stimuli” and type of “stimulation” (F_(1,30)_ = 0.01, p = 0.95, η^2^ < 0.01) were not significant, indicating a comparable performance for CInt and PInt stimuli.

Post-hoc analysis showed a decrease of reaction times induced by anodal tDCS over the mPFC as compared to placebo tDCS in females (RT, 974.3 ± 157.0 ms [mPFC anodal tDCS] *vs.* 1095.9 ± 118.8 ms [placebo tDCS]; p = 0.018), whereas no such effect was observed in males (RT, 1177.0 ± 273.7 ms [mPFC anodal tDCS] *vs.* 1131.7 ± 281.2 ms [placebo tDCS]; p = 0.9). Interestingly, RTs did not differ between males and females in the placebo condition (p = 0.9).

#### Sensations questionnaire

The questionnaires completed by participants at the end of each type of stimulation showed that all of them tolerated the stimulation well and reported only marginal perceptual sensations. Itching and irritation were the most commonly reported perceptual sensations, with light to moderate intensity. Overall, the experienced perceptual sensations started at the beginning of the experiment and did not last long. For each stimulation (real and placebo) and each group (female and male participants), the sensations scores reported during anodal mPFC tDCS were compared with those reported during the placebo tDCS using a Wilcoxon matched pairs test. Both in the males group and in the females group, anodal stimulation over mPFC could not be distinguished from placebo (males: T = 7.0, z = 1.84, p = 0.07; females: T = 7.0, z = 1.18, p = 0.24). Hence, there are no reasons to reject the blinded character of this study on the basis of these results.

### Experiment 2

#### RME data analysis

The overall group reached a mean of 25.3 ± 2.4 points (range = 22–30) indicating age- and gender-adequate ToM abilities according to the Italian normative data provided by Vellante *et al*.^29^.

#### Attribution of Intentions task

Accuracy analysis. No significant effect for the three “groups” (F_(2,45)_ = 0.97, p = 0.39, η^2^ = 0.04), type of “stimuli” (F_(1,45)_ = 0.83, p = 0.37, η^2^ = 0.02), type of “stimulation” (F_(1,45)_ = 1.18, p = 0.28, η^2^ = 0.03), and the interactions between factors were found. A ceiling effect in accuracy for both stimulation conditions was also observed for the females group stimulated with tDCS over Cz (anodal Cz stimulation = 98.0 ± 2.9%; placebo Cz stimulation = 97.2 ± 3.3%, range = 88–100%).

##### Reaction time analysis

RT analysis indicated a significant interaction between the three “groups” and type of “stimulation” (F_(2,45)_ = 4.88, p = 0.001, η^2^ = 0.19; Fig. 1). The type of “stimuli” (F_(1,45)_ = 0.59, p = 0.45, η^2^ = 0.01), the interaction between “groups” and type of “stimuli” (F_(2,45)_ = 1.84, p = 0.17, η^2^ = 0.08) and between type of “stimuli” and type of “stimulation” (F_(1,45)_ = 0.60, p = 0.44, η^2^ = 0.01) were not significant, indicating a comparable performance for CInt and PInt stimuli.

Post-hoc analysis showed no effects of anodal tDCS in the females group that received tDCS over Cz (RT, 1102.0 ± 239.8 ms [Cz anodal tDCS] *vs.* 1148.7 ± 200.5 ms [placebo tDCS]; p = 0.9). A decrease of reaction times during anodal tDCS as compared to placebo tDCS was observed selectively in the females group that received tDCS over the mPFC (p = 0.03), whereas no facilitation effect was observed in males (p = 0.9). Interestingly, RTs recorded during the placebo condition did not differ between the three groups (females group that received tDCS over Cz vs. females group that received tDCS over mPFC: p = 0.9; females group that received tDCS over Cz vs. males group that received tDCS over mPFC: p = 0.9; females group that received tDCS over mPFC vs. males group that received tDCS over mPFC: p = 0.9).

#### Sensations questionnaire

For each stimulation (real and placebo) and each group (males stimulated over mPFC, females stimulated over mPFC and females stimulated over Cz) the sensations scores reported during anodal tDCS were compared with those reported during the placebo tDCS using a Wilcoxon matched pairs test. In the males group, anodal stimulation over mPFC could not be distinguished from placebo (T = 7.0, z = 1.84, p = 0.07). In the females groups, sensations of anodal stimulation over mPFC and over Cz were comparable to placebo stimulation (females mPFC: T = 7.0, z = 1.18, p = 0.24; females Cz: T = 4.0, z = 1.69, p = 0.09). Overall, only few subjects reported low intensity sensations (burning and itching).

## Discussion

In this study, applying a tDCS paradigm, we show for the first time gender-related differences in cognitive ToM ability. In experiment 1, anodal stimulation of the mPFC selectively enhances cognitive ToM in females but not in males. The placebo stimulation is not able to produce the same effect. As suggested by Parkin and colleagues^30^ the use of a control site is recommended in tDCS studies in addition to a placebo condition. Accordingly, to determine whether the tDCS effects reported in Experiment 1 were specifically due to an increase in the activity of the mPFC, and to exclude any unspecific effect of tDCS, we carried out a second experiment. In experiment 2, tDCS stimulation was applied to the vertex (Cz), which served as a control stimulation site. Furthermore, to address site specificity, we follow Nieuwenhuis, Forstmann and Wahenmakers^31^ suggestions and compare the effect of stimulation (anodal and placebo) of the vertex and mPFC in the same analysis. The results of Experiment 2 confirm that neither placebo nor anodal tDCS to the vertex are able to produce the same effect shown in experiment 1. Thus, anodal tDCS over the medial mPFC selectively enhances cognitive ToM performance in females.

We base the interpretation of our results on both domain-specific arguments (i.e., arguments specifically related to the ToM domain) and domain-general arguments (i.e., not specific to the ToM domain). It is important to note that these arguments are not mutually exclusive and probably overlap in the explanation of our results.

As far as domain-general arguments are concerned, one possible non-specific interpretation of our results is *brain excitability*; accordingly, the gender-related differences in cognitive ToM we found could be due to generic gender-related differences in brain excitability on application of tDCS^32^,^33^. Different studies have reported gender-specific effects of tDCS applied to different brain regions, investigating a variety of cognitive abilities, for example to the dorsolateral PFC for verbal working memory^34^, the left parietal cortex for visual spatial attention^35^, the bilateral temporal cortex for somatosensory integration^36^, and the bilateral superior temporal cortex for facial expression recognition^37^. A second possible non-specific interpretation could rely on gender-related *neuroanatomical differences*. Gender-related differences in brain volume and tissue^38^, as well as in structural connectome^39^, in different brain regions, in particular in the ventral^40^ and orbital^41^ frontal cortex, have been described. A recent meta-analysis reported a larger grey matter volume in females, compared to males, in different frontal and prefrontal areas, in particular the middle frontal gyrus, right frontal pole, frontal orbital cortex, and right inferior frontal gyrus, pars triangularis and pars opercularis, as well as a larger grey matter density specifically in the left frontal pole^38^. Using diffusion tensor imaging, Ingalhalikar and colleagues^39^ recently analyzed the diffusion-based structural connectome of a large population of youths and showed that female brains displayed higher interhemispheric connectivity; in particular it was seen mainly in the frontal lobe during adolescence but was more dispersed across the lobes during adulthood. Summing up, the female susceptibility to application of tDCS to the mPFC found in this study could be attributed, at least in part, to both gender-related differences in brain excitability upon application of tDCS and to gender-related neuroanatomical differences in frontal brain regions.

As far as domain-specific arguments are concerned, there are two possible specific interpretations of our findings, i.e., *neurofunctional* and *cognitive strategy* interpretations that, in contrast to the previous arguments, are strictly linked to ToM functioning.

To date, only three neuroimaging studies have investigated neurofunctional gender-related differences in brain activation on different social cognitive tasks, with different outcomes^16^,^42^,^43^. Veroude and colleagues^16^ used a social appraisals task where participants had to indicate whether different phrases described them (“self” condition) and their friend (“other” condition), or judged what their friend would think about them (“reflective” condition); in overall appraisal conditions, the bilateral TPJ was activated to a greater extent in males than in females, whereas no gender-related differences were observed in the mPFC. In contrast, Krach and colleagues^43^ reported gender-related differences in mPFC activation during a ToM task, a ‘Prisoner’s dilemma’ in which participants played against a human or a computer. These authors found that activation of the mPFC when playing against a human was larger for males compared with females. More recently, Frank and colleagues^42^ investigated gender-related differences in ToM, using ToM stories with a second-order false-belief task, compared to (a) stories requiring only pragmatic reasoning but no ToM, and (b) unlinked sentences (control condition) not requiring ToM or pragmatic reasoning. Comparing ToM stories with control condition these authors found greater activation of the left mPFC and the left TPJ, and greater deactivation of the bilateral ventromedial PFC in females than males. Interestingly, according to these authors, females may use strategies related to perspective-taking more than males, along with the use of ToM reasoning more often than males during the comprehension of stories requiring only pragmatic reasoning but no ToM. Moreover, as far as deactivation of the ventromedial PFC is concerned, the authors suggest that females may have less difficulty than males in disengaging from self-referencing or internally directed thoughts during ToM reasoning.

*Cognitive strategy* interpretation relies mainly on results reported by Blakemore and colleagues, who compared ToM performances between adolescents and young adults^44^. Different developmental neuroimaging studies^44^,^45^,^46^ showed striking consistency with respect to the direction of change in mPFC activity related to ToM ability: the involvement of the mPFC during ToM tasks decreases with transition from adolescence to adulthood. Although the same neural network is active during ToM performances, the relative roles of the different areas change with age, with activity moving from the anterior (mPFC) regions to posterior (TPJ) regions from adolescence to adulthood. These findings were found both in the ventromedial PFC, comparing affective and cognitive ToM^47^, and in the dorsolateral PFC, using scenarios requiring intentional causality attribution (involving intentions and consequential actions) and physical causality attribution (involving natural events and their consequences)^48^. According to Blakemore^44^, one possible explanation is that the cognitive strategy for ToM changes from adolescence to adulthood; in particular, ToM in adults may be more automatic than in adolescents, who instead might base their judgments on novel computations performed in the mPFC. It is worth noting that almost all of these studies involved only females^45^,^48^ or only males^47^, and to date there are no neuroimaging studies that specifically analyze gender-related differences in ToM during this developmental transition. For this reason, we cannot exclude the existence of gender-related differences both during the developmental transition, with respect to the timing, and in the outcome of this transition, with respect to cognitive strategies. In particular, the outcome of the developmental transition from adolescence to adulthood may be different between females and males, with differential recruitment of the mPFC leading to different ToM cognitive strategies. Thus, our results could be attributed to these gender-related differences at both cognitive and neural levels.

### Limitations

There are limitations of our study that need to be acknowledged. Generalization of the present study is limited as only one task was applied during tDCS stimulation, and a ceiling effect was observed on the accuracy data. The relative small number of subjects and the lack of a control task condition represent further potential limitations. Furthermore, an important discussion surrounding tDCS research is related to how reproducibility of reported effects should be evaluated^49^. A recent review^50^ on the effects of tDCS on social cognition pointed out that some studies produced results inconsistent to each other, likely due to methodological differences (e.g., intensity and duration of the stimulation, online vs. offline stimulation, electrode size, scalp placement, study design, experimental task). Therefore, more research, based on larger samples, different tasks, and including population of patients with ToM deficit, is needed to elucidate the reasons for these discrepancies and to verify whether the observed tDCS-induced changes in social cognition are maintained across studies.

## Conclusion

Despite these limitations, the findings of the present study are relevant for at least three main reasons. First, using brain stimulation we found preliminary evidence of gender-related differences in cognitive ToM, extending previous findings almost exclusively concerning the affective dimension of social cognition. Second, our results suggest that these differences emerge with the anodal stimulation of the anterior part of the distributed neural network underpinning ToM, i.e., the mPFC, confirming the crucial role of this area in cognitive ToM. Third, more in general, our results show that taking into account gender-related differences is recommended for the investigation of social cognitive processes involving ToM, in particular using brain stimulation techniques.

Furthermore, it is now recognized that tDCS can improve social cognition^46^ and can be used for the treatment of neuropsychiatric disorders^51^. Hence, it is crucial to understand the effects of this neuromodulatory technique on cognitive ToM, not only for the comprehension of the gender-related mechanisms underlying ToM but also for the potential contribution of these findings to the development of effective non-invasive brain stimulation treatments in patients with different neuropsychiatric disorders^52^, in particular those characterized by ToM impairment.

## Methods

This study was approved by the ethics committee of the IRCCS Centro San Giovanni di Dio Fatebenefratelli, Brescia, Italy, and was conducted in accordance with the tenets of the Declaration of Helsinki.

### Experiment 1

#### Participants

Thirty-two healthy Italian native speakers volunteers were recruited (16 females and 16 males; age range = 18–31 years; mean age = 23.6 ± 3.6 years; mean education = 14.7 ± 2.6 years) with the following inclusion criteria: age between 18 and 35 years; no history of neurological, psychiatric diseases or substance-related disorders, no significant general medical condition; no contra-indication to tDCS. All the participants were right-handed as assessed with the Edinburgh Handedness Inventory^53^. All participants were informed about the procedures and the possible risks associated with tDCS and provided their written informed consent after a safety screening.

#### Theory of mind tasks

All the participants performed two ToM tasks: the Reading the Mind in the Eyes (RME), to assess individual ToM abilities, and the Attribution of Intentions (AI) task, to test the effect of tDCS on cognitive ToM ability.

The RME is an advanced ToM task involving presentation of photographs of the eye region of human faces, and evaluates the subject’s ability to represent others’ mental states by observing only their eyes^54^. Participants are required to choose which word, among four options, best describes what the character in the photograph is thinking or feeling. Each participant was shown photographs on a computer screen and responded orally. The total number of correct choices represented the RME score. Participants were tested on RME before to be involved in the tDCS brain stimulation to exclude participants with subtle ToM difficulties^54^.

**Figure 2:**
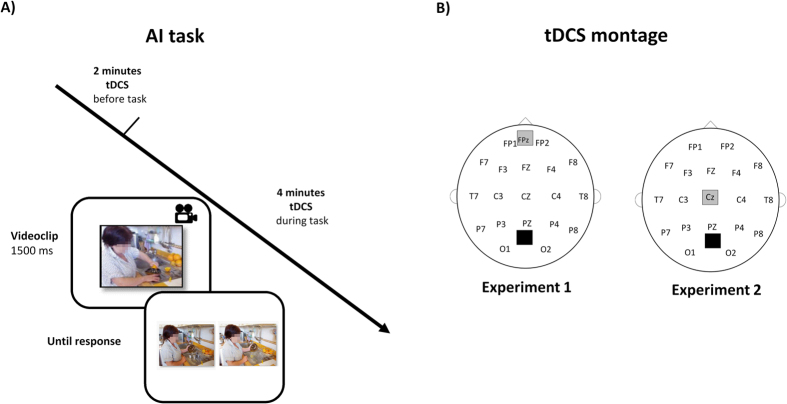
(**A**) Experimental design. Anodal or placebo tDCS was applied 2 minutes before the beginning of the experimental block and covered the entire AI task. In the AI task, a short video was played and then the participant’s task was to choose the picture showing the logical story ending, by pushing one of the two buttons on a button box. (**B**) Schematic drawing of electrode positions.

The AI task is a novel video version of a cognitive ToM task previously used in a comic strips form^55^,^56^,^57^,^58^. The AI task was a story completion task in which participants were asked to demonstrate their comprehension of the stories by choosing the most appropriate story endings. Each story consisted of one short video (development phase), followed by a choice between two concluding pictures (response phase). The response phase presented two possible story endings with two answer pictures showed simultaneously till the participant responded pressing the corresponding button on the button box as fast as possible: the correct picture represented a probable and congruent effect, whereas the incorrect picture represented an improbable or incongruent effect (Fig. 2A).

The AI task included two types of videoclips: Private intention (PInt) and Communicative intention (CInt). In the PInt videoclips, participants were required to recognize another person’s intention based on the observation of that person’s isolated action, e.g., hanging a picture on the wall; in the CInt videoclips, participants were required to recognize another person’s communicative intention based on the observation of a social interaction, e.g., observing a person obtaining a glass of water by asking another person to get it for her. The present study included 34 short video stories for each of the two types of stimuli (PInt and CInt), for a total of 68 stories.

#### tDCS procedure

Participants were seated in a dimly lit room facing a computer monitor placed at a distance of 60 cm. The stimuli were presented using Presentation software (Version 16.3, www.neurobs.com) running on a personal computer with a 15-inch screen and the participants’ responses were collected with a button box. Visual location of the correct answer on the screen was randomized.

The items were divided into two blocks (17 PInt and 17 CInt each) that were designed for the two types of stimulation (anodal and placebo stimulation). The category of stimuli and visual complexity of the scenes were matched and counterbalanced between the two experimental sessions. Moreover, four additional stimuli were selected and used for a training session (2 PInt and 2 CInt). Each experimental block took about 4 minutes to complete. Accuracy was recorded as the number of correct trials. Reaction Times (RT) to each correct response was recorded in millisecond (ms) from the onset of the two concluding pictures presentation until the response was detected. Anodal tDCS was applied using a battery-driven, constant-current stimulator (BrainStim, EMS, Bologna, Italy) through a pair of saline-soaked sponge electrodes (7 cm × 5 cm). A constant current of 1 mA was applied for 6 minutes (a ramping period of 10 seconds both at the beginning and at the end of the stimulation), starting 2 minutes before the beginning and covering the complete task. The current density (0.029 mA/cm^2^) was maintained below the safety limits^59^. To guarantee a stable placement of electrodes on the scalp we used cross elastic bands under the chin for all participants. To achieve a consistent and appropriate amount of contact medium under the electrodes and avoid oversaturation of sponges one saline-filled syringe (5 ml) per sponge per session was applied for each participants and to reduce contact impedance an electroconductive gel was applied below the electrodes.

The study was a randomized double-blind experiment: the participants and the experimenter did not know which stimulation was delivered. The two stimuli sessions corresponded to two stimulation conditions: anodal mPFC stimulation and placebo stimulation. The stimulation conditions were randomized across participants and executed on two consecutive days at the same day-time to minimize the likelihood of confounding interference effects. The 50% of male and female participants received anodal stimulation on day 1 and placebo stimulation on day 2, while the 50% of male and female participants received placebo stimulation on day 1 and anodal stimulation day 2. Placebo or anodal tDCS were delivered after entering a number code to the device and this step allows blinding of the operator before and during tDCS administration.

Our choice of the mPFC for stimulation was based on the findings of our previous fMRI studies (reference MNI coordinates: 0, 60, 18; see refs 55, 56, 57, 58 in which the activation of this area was recognized as pivotal in intention processing, a key cognitive ToM ability. In order to stimulate the mPFC, the anode was placed over the Fpz and the cathode was placed between the inion and the Oz (Fig. 2B), according to the 10–20 EEG international system for electrode placement^60^. Specifically, an EEG cap was gently secured on the head of each subject and positioned with Cz at the vertex, as measured using surface anatomical landmarks, and defined as the intersection of the nasion–inion and interaural lines; subsequently Fpz was marked with a pencil and identified as the center of the anode. The EEG cap was then removed to allow tDCS montage.

In the placebo stimulation, the tDCS procedure was the same but the current was turned off 10 seconds after the beginning of the stimulation (plus the duration of the fade-in and fade-out periods = 10 seconds) and was turned on for the last 10 seconds of the stimulation period. Therefore, the participants experienced an itching sensation below the electrodes at the beginning and end of the stimulation, making this condition indistinguishable from the experimental stimulation^61^.

In order to detect differences in the perception of sensations, to blind the participants to the type of stimulation they were receiving, and to register potential side effects of tDCS, at the end of the stimulation session we asked the participants to answer a sensations questionnaire about the perceptual sensations experienced during anodal and placebo tDCS^62^.

#### Statistical analyses

Statistical analyses were performed using Statistica software (version 10; www.statsoft.com). Demographic variables and performances on the RME test were compared between males and females using non-parametric (Mann-Whitney test) analyses. AI task performances (accuracy and RTs) were analyzed using repeated-measures Analysis of Variance (ANOVA). Considering that the RT data were not normally distributed (Kolmogorov-Smirnov Test: d = 0.10, p < 0.01; Skewness +1.0, right skewed), we adopted logarithmic transformation of data and analyzed log-transformed RTs. A repeated-measures 2 × 2 × 2 ANOVA was performed, which included as factors two types of “stimulation” (anodal mPFC or placebo, within participants), two types of “stimuli” (PInt and CInt, within participants), and two “gender” groups (female or male, between participants). Post-hoc analysis was carried out using the Bonferroni correction for multiple comparisons. Sensation scores were compared between anodal tDCS and placebo tDCS, separately for females and males groups using Wilcoxon matched pairs test. Statistical significance was set at p < 0.05.

### Experiment 2

#### Participants

Sixteen healthy females Italian native speakers matching the demographic variables of the participants enrolled in experiment 1 were enrolled in experiment 2 (age, 22.8 ± 1.6 years; education, 15.1 ± 1.9 years). All the participants resulted to be right-handed on the Edinburgh Handedness Inventory^53^. Participants in Experiment 1 did not take part in Experiment 2. The inclusion criteria, experimental procedure, and materials used were the same as those described in Experiment 1.

#### Procedure

The RME task, the AI task, the sensations questionnaire, and the stimulation parameters were identical to those used in Experiment 1, except for the anode, which was placed on the vertex (Cz) according to the EEG 10–20 international system (Fig. 2B).

#### Statistical analyses

AI task performances (accuracy and RT) were analyzed using an ANOVA analysis. Considering that the RT data were not normally distributed (Kolmogorov-Smirnov Test: d = 0.10, p < 0.01; Skewness +1.0, right skewed), we adopted logarithmic transformation of data and analyzed log-transformed RTs. A repeated-measures 2 × 2 × 3 ANOVA including two types of “stimulation” (anodal or placebo, within participants), two types of “stimuli” (PInt and CInt, within participants) and three “groups” (male mPFC of experiment 1, female mPFC of experiment 1 and female Cz of experiment 2, between participants) was performed. Post-hoc analysis was carried out using the Bonferroni correction for multiple comparisons. Sensation scores were compared between anodal tDCS and placebo tDCS, separately for each experimental group using the Wilcoxon matched pairs test. Statistical significance was set at p < 0.05.

### Ethics statement

All procedures performed in studies involving human participants were in accordance with the ethical standards of the institutional research ommittee. Informed consent was obtained from all individual participants included in the study. Ethics approval was obtained from the local Ethical Committee (IRCCS Centro San Giovanni di Dio Fatebenefratelli, Brescia, Italy).

## Additional Information

**How to cite this article:** Adenzato, M. *et al*. Gender differences in cognitive Theory of Mind revealed by transcranial direct current stimulation on medial prefrontal cortex. *Sci. Rep.* **7**, 41219; doi: 10.1038/srep41219 (2017).

**Publisher's note:** Springer Nature remains neutral with regard to jurisdictional claims in published maps and institutional affiliations.
